# Zinc‐Catalysed Depolymerization of Poly(Butylene Succinate) and Poly(Butylene Adipate‐*co*‐Terephthalate) and Enhanced Degradation of Catalyst‐Polymer Composite Films

**DOI:** 10.1002/cssc.202502332

**Published:** 2025-12-01

**Authors:** Fannie Burgevin, Jack A. Stewart, Annie May, Matthew J. Cullen, Antoine Buchard, Matthew G. Davidson, Matthew D. Jones

**Affiliations:** ^1^ Department of Chemistry University of Bath Bath UK; ^2^ Institute of Sustainability and Climate Change University of Bath Bath UK; ^3^ Department of Chemistry Green Chemistry Centre of Excellence University of York York UK

**Keywords:** biodegradable polymers, depolymerization, homogeneous catalysis, polymers, sustainable chemistry

## Abstract

The depolymerization of poly(butylene succinate) (PBS) and poly(butylene adipate‐*co*‐terephthalate) (PBAT) with a highly active zinc catalyst was investigated. The methanolysis of PBS in solution was optimised by varying temperature, catalyst loading, and methanol equivalents, giving a maximum conversion of 98% after 48 h with a dimethyl succinate yield of 62%. Solvent‐free methanolysis of PBS and PBAT was shown to reach high conversion after 1 h, although increased temperature was required (100–130°C). When the catalyst was embedded into thin films of PBS and PBAT, a significant loss of mass and a reduction in molecular weight was observed after incubation at 50°C in methanol, comparing favorably with samples of pure polymer. Some increase in degradation activity was also observed in deionized water. This work demonstrates the application of common chemical recycling techniques to increasingly relevant bio‐derived polyesters as well as the potential for embedded zinc catalysts to promote degradation.

## Graphical abstract

Biodegradable polymers are a crucial part of a sustainable future with plastics. The depolymerization of two prominent examples, poly(butylene succinate) and poly(butylene adipate‐*co*‐terephthalate), is reported with a highly active zinc catalyst. This includes rapid depolymerization in neat methanol. The catalyst was also embedded into polymer films and incubated in water or methanol, showing significant mass loss and reduction in molecular weight compared to a pure control sample.

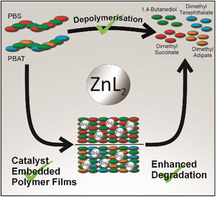

## 1 Introduction

Plastics are an incredibly useful and versatile class of materials that have become ubiquitous throughout the world [1]. However, the sheer volume of plastic that has been produced has led to catastrophic levels of plastic pollution across almost all ecosystems and trophic levels [2, 3, 4, 5, 6, 7, 8]. Many petrochemically derived plastics degrade into microplastic particles in the environment, which can bioaccumulate and have been shown to be toxic [9, 10, 11, 12]. Solving this problem will require an interdisciplinary approach involving the reduction of plastic use through legislation [5, 13, 14], improved methods of reusing and recycling [15, 16, 17, 18, 19], and the development of sustainable polymers that can be easily degraded to benign products [20, 21, 22].

One example of an emerging biodegradable polymer is poly(butylene succinate) (PBS). PBS is an aliphatic polyester with promising physical properties that are comparable to polyethylene and polypropylene in terms of melting point, toughness, glass transition temperature (*T*_g_), tensile strength, and toughness [23]. PBS is typically synthesized through a two‐step polycondensation reaction between 1,4‐butanediol (1,4‐BDO) and succinic acid with chain extenders required to produce processable polymer of around 40,000 gmol^−1^ [24]. The ring‐opening polymerization of butylene succinate lactones has also been used to access high molecular weight PBS [25]. PBS can be useful for many applications, including the packaging and biomedical industries [26, 27]. There are also several examples of useful materials based on PBS such as blends, composites, and copolymers, which seek to augment the attractive properties of PBS whilst offsetting disadvantageous elements, such as brittleness [23, 24, 26, 28].

The degradability of PBS in the natural environment is crucial for its usefulness as a replacement for hydrocarbon‐based plastics. Some studies for degradation and biodegradation of PBS have shown limited signs of degradation in soil [29] and slow biodegradation in industrial compost [23, 30, 31]. Although biodegradation is extremely important for the inevitable leakage of plastic into the environment, it remains important to consider end‐of‐life options that retain the value of the material. Mechanical recycling is an important end‐of‐life option, and it is widely used for commodity plastics such as poly(ethylene terephthalate) (PET), although there are issues around contamination and the gradual loss of mechanical properties over multiple cycles [32]. A recent report from Muniyasamy et al. tested a number of mechanical properties of several polymers over seven reprocessing steps [33]. For PBS, tensile strength was maintained over seven cycles, but other parameters, such as strain at break, impact strength, and melt flow index were diminished. Incarnato et al. showed that the recyclability of poly(lactic acid) (PLA)/PBS blends was enhanced by increasing levels of PBS [34]. This shows that mechanical recycling is possible for PBS, but issues could be encountered when blended or composited. Another approach, such as chemical recycling, may therefore be more favorable.

The chemical recycling of polymers occurs when waste plastic is broken down into repolymerizable monomers (depolymerization) or valuable small molecules (degradation or upcycling). This is a crucial process for the development of a circular economy for polymers [19, 35, 36, 37, 38]. The alcoholysis of polyesters is one of the most important areas of chemical recycling with examples such as PET glycolysis [39, 40, 41] and PLA methanolysis [42, 43, 44, 45, 46, 47, 48, 49, 50, 51, 52, 53] among the most well‐studied. There are limited reports of PBS alcoholysis including from Yang et al. who depolymerised a range of polymers with Zn(HMDS)_2_ [54]. PBS reached high conversion to 1,4‐BDO and dimethyl succinate (DMS) after 34 h in CH_2_Cl_2_ at 50°C or 9 h in toluene at 100°C. A different approach was taken by Wu and coworkers who transformed a range of succinate‐based polymers into N‐substituted succinimides in a succinimide‐based ionic liquid [55]. After an hour at 130°C, a range of succinimide derivatives could be produced in high yield from PBS and the relevant amine. The upcycling of end‐of‐life PBS through vitrimerisation has also been reported [56]. The cross‐linking process retained many properties of the original polymer whilst improving melt strength and stress relaxation capabilities and making the material less soluble.

Another commonly used biodegradable polyester is poly(butylene adipate‐*co*‐terephthalate) (PBAT). PBAT shares the 1,4‐BDO comonomer with PBS, which can be produced bio‐catalytically from carbohydrate feedstocks [57]. PBAT is an example of an aromatic copolyester that has good biodegradability alongside some properties that are similar to low‐density polyethylene, such as tensile strength, elongation at break, and melt flow index [58]. These properties are ideal for film blowing applications and PBAT can be blended with other polymers, such as PLA, to facilitate this process [59]. PBAT is made through the polycondensation of 1,4‐BDO, adipic acid and terephthalic acid with physical properties and biodegradability controlled by the ratio of monomers. PBAT has been shown to degrade in certain environmental conditions, much more effectively than other aromatic polyesters, such as PET [60, 61, 62]. However, there is a trade‐off between reduced biodegradability and improved thermal characteristics when increasing the terephthalate content [58]. To enhance degradability whilst improving the physical properties of PBAT, it is often blended with other materials such as starch and PLA. PBAT and its composites have found applications in packaging, mulch films, and carrier bags [58, 63]. Some studies have investigated the mechanical recycling of PBAT and found that the polymer undergoes chain scission reactions leading to reduced physical properties [64, 65].

Wang and coworkers demonstrated the chemical recycling of PBAT with high conversion reached after 12 h at 100°C in methanol with a Zn(HMDS)_2_ catalyst [54]. The sequential chemical recycling of PBAT with PLA was reported and the three products were isolated at high yields. High‐temperature hydrolysis (170°C) of PBAT in the presence of an adipic acid catalyst has been reported [66]. Interestingly, the final reaction mixture could be directly repolymerized to give fresh PBAT with properties similar to that of the commercial polymer. The hydrolysis of PBS and PBAT using dizinc catalysts in alkaline solution has also been reported [67]. Research has been conducted into the upcycling of waste poly(butylene terephthalate) (PBT) into PBAT through a cyclo‐depolymerization followed by copolymerization with poly(butylene adipate) diols [68, 69]. A recent report from Wang et al. extended this idea to include a flame‐retardant chain segment, therefore creating a biodegradable polymer with flame‐retardant properties from a nondegradable waste material [70]. Not only were several key physical properties, such as elongation at break, tensile strength, yield strength, and elastic modulus enhanced, biodegradability was also significantly increased. This suggests a promising method for increasing the utility of already more sustainable polymers.

One method for assisting the depolymerization, or biodegradation, of a plastic product is to introduce an active material into the polymer structure. ZnO nanoparticles are often added to plastic products to impart antimicrobial properties. Liu et al. studied the effect of ZnO nanoparticles on the properties of PBAT [71]. They found that increasing ZnO content improved the biodegradability of the material, although physical properties were affected. The incorporation of ZnO has also been shown to accelerate the disintegration of a commercial PLA/PBAT blend in a composting environment [72]. Luo et al. blended PLA and PBAT with FeCl_3_ to give a material with good physical properties and increased biodegradation compared to PLA [73]. Another approach is to utilize additives found in plastic to facilitate selective degradation to useful products. A recent example from Stache and coworkers demonstrated the upcycling of poly(vinyl chloride) (PVC) and polystyrene (PS) under UV irradiation [74]. Niu and coworkers embedded a dizinc esterase mimic complex into PBAT [75]. Using a PBAT film with 5 wt% embedded catalyst, a 20% yield of 1,4‐BDO was produced in aqueous solution compared to a 1% 1,4‐BDO yield for the pure polymer. An improvement in some physical properties was observed upon blending with catalyst and biocompatibility was maintained. Results suggested that the catalyst in the solid state was the active component, although leaching of zinc complex into solution was noted.

Herein, we report a systematic study of PBS depolymerization with a highly active zinc (II) depolymerization catalyst that we have previously reported [76]. The reaction temperature and concentration of methanol and catalyst are varied and solvent‐free depolymerization in neat methanol is investigated. The degradation behavior of catalyst‐embedded thin films in methanol and water is reported. The aromatic copolyester, PBAT, is also tested for depolymerization in methanol and in thin films. The application of chemical recycling techniques to these polymers while using mild conditions satisfies many principles of green chemistry including the prevention of waste, use of benign solvents, catalysis, and inherently benign chemistry. Moreover, the embedding of catalyst within polymer films is an excellent example of a material designed for degradation.

## 2 Results and Discussion

### 2.1 PBS Methanolysis in THF

**SCHEME 1:**
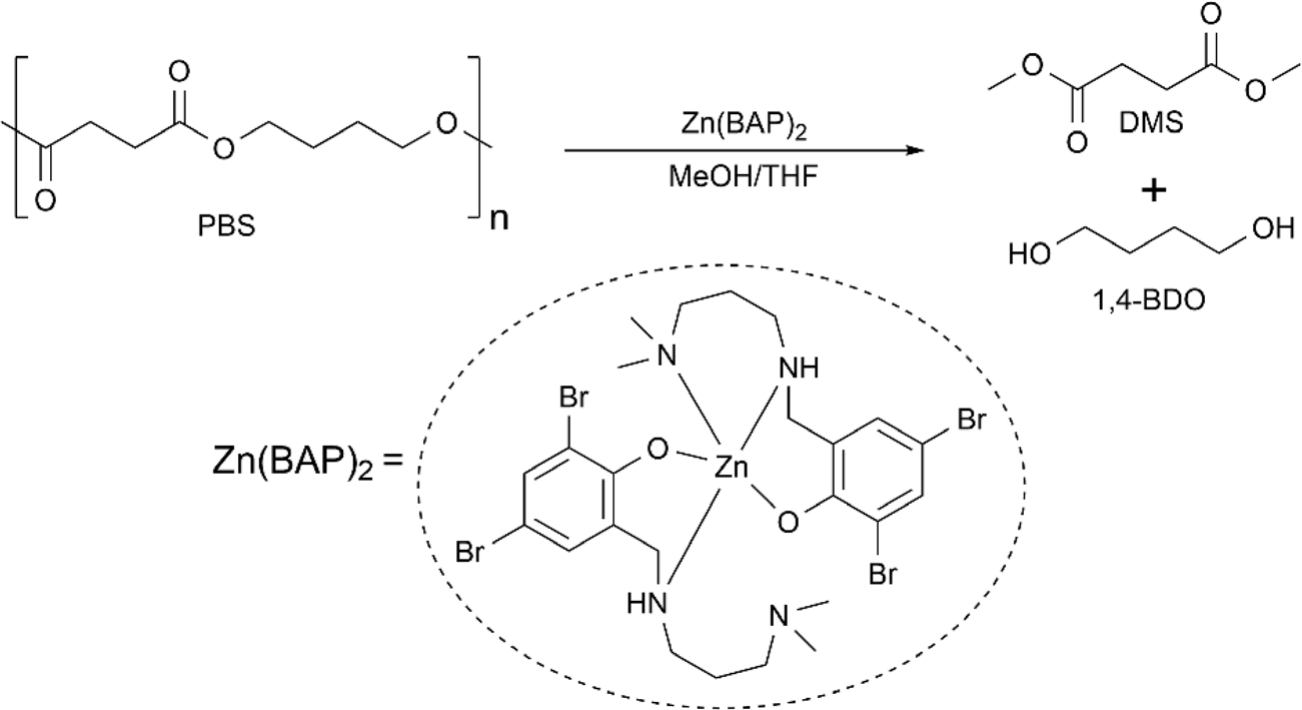
Depolymerization of PBS to 1,4‐BDO and DMS and the structure of Zn(BAP)_2_.

Initial experiments focused on the methanolysis of PBS pellets (M_*n*_(SEC) ≈ 70,000 gmol^−1^) to 1,4‐BDO and DMS in THF (Scheme 1). A previously reported dibromo amino‐phenolate zinc (II) catalyst, referred to herein as Zn(BAP)_2_, was chosen for this reaction as it is air‐stable and highly active for the depolymerization of PLA, PET, and BPA‐PC [76, 77]. PBS, catalyst, and products were all fully soluble in the THF/MeOH reaction mixture. Therefore initial conditions could be chosen using the literature of zinc‐catalysed solution‐phase polyester methanolysis as a guide (80°C, 8 wt% catalyst, 10 equiv. MeOH, 4 mL THF) [51, 52, 53, 78]. The methanolysis of PBS, however, was considerably slower than expected from PLA methanolysis, which was complete in 90 min under comparable conditions [76]. This compares to 95% PBS conversion after 48 h, despite both PBS and PLA being linear, aliphatic polyesters.

**FIGURE 1:**
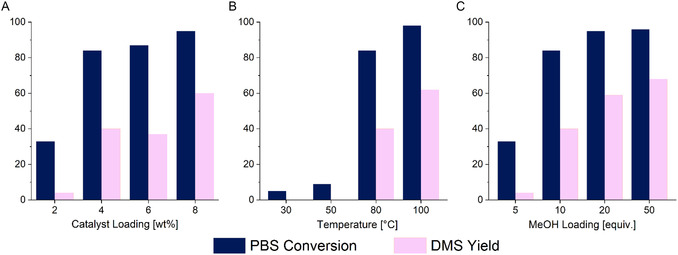
Optimisation of PBS methanolysis in THF. (A) Catalyst loading comparison. Conditions: PBS pellets (0.25 g), 2–8 wt% Zn(BAP)_2_ (5–20 mg, 0.4–1.7 mol% relative to polymer repeat unit), 10 equiv. MeOH relative to polymer repeat unit (0.6 mL), 4 mL THF, 80°C, 48 h. (B) Temperature comparison. Conditions: PBS pellets (0.25 g), 4 wt% Zn(BAP)_2_ (10 mg, 0.9 mol% relative to polymer repeat unit), 10 equiv. MeOH relative to polymer repeat unit (0.6 mL), 4 mL THF, 30–100°C, 48 h. (C) Methanol loading comparison. Conditions: PBS pellets (0.25 g), 4 wt% Zn(BAP)_2_ (10 mg, 0.9 mol% relative to polymer repeat unit), 5–50 equiv. MeOH relative to polymer repeat unit (0.3–3.0 mL), 4 mL THF, 80°C, 48 h.

A series of optimisation experiments were conducted focusing on temperature, catalyst loading, and methanol loading of PBS methanolysis (Figure 1A, Table S1). Wang et al. demonstrated that the O‐CH_2_^1^H NMR signals of the succinate subunits differed subtly between free diacid and the mono‐ and disuccinate esters with 1,4‐BDO [54]. The equations detailed in Figure S4 allowed for the calculation of PBS conversion (*X*_PBS_), selectivity to DMS (*S*_DMS_), and DMS yield (*Y*_DMS_) from the relative integrations of these signals. This is similar to the established quantification of PLA methanolysis through integration of the methine region of the ^1^H NMR spectra [42]. Using 4 wt% catalyst, 84% conversion was achieved after 48 h with a selectivity and yield of DMS of 47% and 40%, respectively. The moderate selectivity in this reaction suggests that there are short‐chain oligomers present with terminal succinate methyl ester groups. There was a minimal difference in activity when the catalyst loading was increased to 6 wt%, and the yield was slightly reduced (*X*_PBS_ = 87%, *S*_DMS_ = 43%, *Y*_DMS_ = 37%). However, when the loading was further increased to 8 wt%, a conversion of 95% was attained alongside a DMS yield of 60%. A catalyst loading of 2 wt% led to a significant reduction in conversion and yield (*X*_PBS_ = 33%, *S*_DMS_ = 13%, *Y*_DMS_ = 4%).

No DMS was produced at 30 and 50°C after 48 h and only 5% and 9% of PBS was converted (Figure 1B, Table S2). Increasing the temperature from 80 to 100°C resulted in a significant increase in yield with almost full conversion reached over 48 h (80°C: *X*_PBS_ = 84%, *S*_DMS_ = 47%, *Y*_DMS_ = 40%; 100°C: *X*_PBS_ = 98%, *S*_DMS_ = 63%, *Y*_DMS_ = 62%).

The reaction required a minimum of ten molar equivalents of methanol to reach reasonable conversion after 48 h (5 equiv. MeOH: *X*_PBS_ = 33%, *S*_DMS_ = 12%, *Y*_DMS_ = 4%; 10 equiv. MeOH: *X*_PBS_ = 84%, *S*_DMS_ = 47%, *Y*_DMS_ = 40%) (Figure 1C, Table S3). Activity increased significantly when loading was doubled to 20 equivalents giving a conversion and yield of 95% and 59% respectively. A further increase in DMS yield was observed with 50 equivalents of methanol, however, conversion of PBS was relatively unchanged (50 equiv. MeOH: *X*_PBS_ = 96%, *S*_DMS_ = 71%, *Y*_DMS_ = 68%).

### 2.2 Solvent‐Free Methanolysis of PBS and PBAT

The depolymerization of PBS in neat methanol was tested whilst modifying temperature and the loading of Zn(BAP)_2_. The removal of THF from this process enhances sustainability through minimising waste and toxicity whilst reducing the volume of the reaction relative to the initial mass of polymer. In this case, ‘solvent‐free’ refers to the outset of the reaction where PBS or PBAT are insoluble and thus react heterogeneously. However, a homogeneous solution is formed upon production of the products. After 1 h at 130°C, no conversion was observed in the absence of a catalyst, in keeping with the PLA literature (Table 1) [53]. The introduction of 2 wt% Zn(BAP)_2_ gave high conversion and moderate yield after 1 h (*X*_PBS_ = 87%, *S*_DMS_ = 66%, *Y*_DMS_ = 57%) and the results were essentially unchanged at 4 wt% loading (*X*_PBS_ = 91%, *S*_DMS_ = 63%, *Y*_DMS_ = 57%). Further increasing the loading to 8 wt% resulted in full conversion after 1 h with a selectivity and yield of DMS of 86% and 85% respectively. This compares Favorably with the Zn(HMDS)_2_‐catalysed methanolysis of PBS reported by Yang and coworkers, who reported PBS conversion and DMS yield of 90% and 83% after 9 h in toluene at 100°C using a similar catalyst loading (Zn(HMDS)_2_ = 2 mol%, Zn(BAP)_2_ = 2.6 mol%) [54].

**TABLE 1:** Methanolysis of PBS and PBAT in neat alcohol with Zn(BAP)_2_.^a^

Poly.	Alcohol	Time [h]	Loading [wt%]	T [°C]	*X*_PBS_ b [%]	*Y*_Diester_ b [%]	*X*_PBA_ b [%]	*X*_PBT_ b [%]	*Y*_DMT_ b [%]
PBS	MeOH	1	0	130	<1	<1	–	–	–
PBS	MeOH	1	2	130	87	57	–	–	–
PBS	MeOH	1	4	130	91	57	–	–	–
PBS	MeOH	1	8	130	100	85	–	–	–
PBS	MeOH	1	4	100	85	35	–	–	–
PBS	MeOH	1	4	80	46	10	–	–	–
PBS	EtOH^c^	2	8	130	88	27	–	–	–
PBS	*n*‐BuOH^d^	2	8	130	51	0	–	–	–
PBAT	MeOH	1	8	130	–	–	84	94	87
PBAT	EtOH^c^	2	8	130	–	–	<1	<1	<1
PBAT	n‐BuOH^d^	2	8	130	–	–	<1	<1	<1

a Conditions: PBS/PBAT pellets (0.25 g), 0–8 wt% cat. loading (0–20 mg, 0–3.9 mol% relative to polymer repeat unit), methanol (2 mL, 33 equiv. relative to polymer repeat unit);

b Calculated from ^1^H NMR spectroscopy;

c Ethanol (2.78 mL, 33 equiv. relative to polymer repeat unit);

d n‐Butanol (4.38 mL, 33 equiv. relative to polymer repeat unit).

Reducing the temperature had a more notable impact on DMS yield than adjusting the catalyst loading. At 100°C, a DMS yield of 35% was obtained and this dropped to 10% after 1 h at 80°C, the latter coinciding with a PBS conversion of 46%. These results contrast to a recent report on solvent‐free PLA methanolysis with an imino‐pyrrole zinc complex where the reaction proceeded rapidly at reduced temperatures but was considerably slowed down by catalyst loadings below 4 wt% [53]. When the most active conditions for PBS methanolysis were applied to PBAT, conversions of 84% and 94% were recorded for the poly(butylene adipate) and poly(butylene terephthalate) units, respectively, alongside a dimethyl terephthalate (DMT) yield of 87%. This result suggests that the aromatic component of PBAT does not drastically affect the depolymerization when compared with PBS. This is significantly quicker than the Zn(HMDS)_2_ catalysed depolymerization of PBAT in toluene, which took 9 h to reach similar conversion and yields [54]. We have previously demonstrated catalyst recycling using an imino‐pyrrole zinc (II) complex for PLA methanolysis in similar conditions through the removal of the volatile product by distillation. The products of PBS depolymerization have boiling points in excess of 200°C and so the temperature required for distillation would likely deactivate the catalyst. However, this does suggest that the catalyst would still be active if separation were achieved [53].

PBS and PBAT were subsequently tested for neat alcoholysis with ethanol and n‐butanol to expand the product scope and provide comparison with PLA literature [52, 53]. For PBS, reactivity decreased with alcohol size, as expected. Ethanolysis yielded 28% diethyl succinate with 88% conversion of PBS. The reaction with n‐butanol converted 51% of polymer but failed to produce the target product. Interestingly, PBAT did not react under these conditions despite fully solubilizing in the reaction mixture at 130°C.

### 2.3 Degradation of PBS Films in Methanol and Water

As previously discussed, Niu et al. recently reported a dizinc catalyst that was active for the hydrolysis of PBAT with solvated catalyst [67] and embedded in the solid polymer matrix [75]. With the activity of Zn(BAP)_2_ for PBS and PBAT methanolysis established in solution, the effect of the behavior of catalyst‐embedded thin films in methanol at 50°C was investigated. This follows the principle of designing for degradation wherein all aspects of the lifetime of a material should be considered from the outset.

**FIGURE 2:**
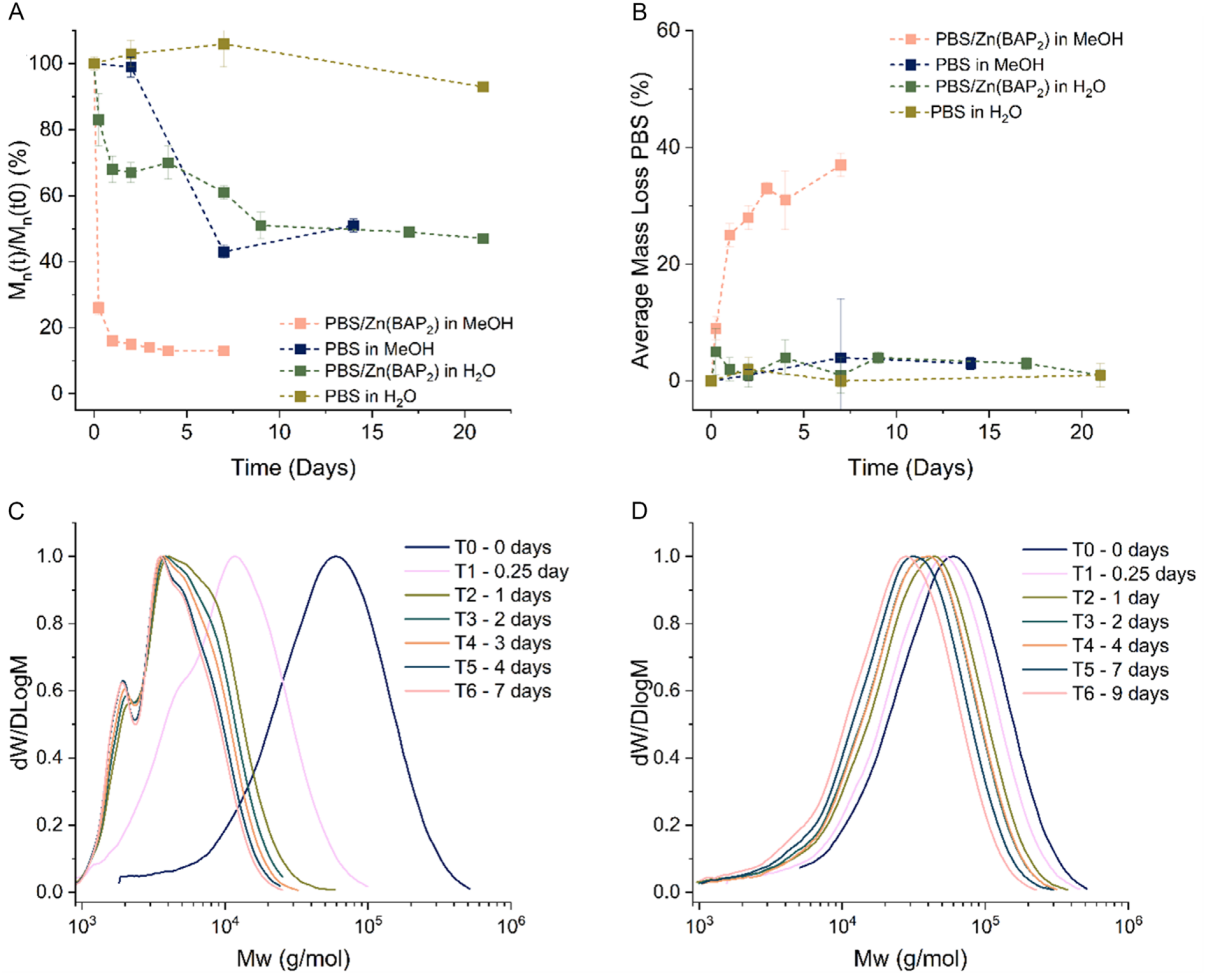
(A) Average % mass loss of PBS after incubation in water and methanol at 50°C, (B) Average molar mass of PBS relative to average initial molar mass over time in methanol and water at 50°C, (C) SEC traces of PBS with catalyst embedded over time at 50°C in methanol, and (D) SEC traces of PBS with catalyst embedded over time at 50°C in methanol.

The degradation of PBS films embedded with 4 wt% catalyst and films without catalyst was monitored over time by mass loss and change in molar mass. A higher mass loss was clearly observed for catalyst‐embedded PBS films in methanol with an average of 37 ± 3% mass loss measured after 7 days (Figure 2A), whereas 1% mass loss was observed without catalyst in the same conditions. A rapid decrease in molar mass was also observed, reaching an average of 13 ± 1% of the initial molar mass after 7 days. The degradation was slower for films not containing catalyst (Figure 2B), suggesting the catalyst was able to accelerate the degradation of thin films in methanol.

The impact of the embedded catalyst was also investigated in deionized water. The degradation was slower than in methanol with only 1% mass loss measured after 21 days of incubation, but the molar mass reached an average of 47 ± 2% of the original molar mass (Figure 2A,B). In comparison, very limited change in molar mass was observed for films without catalyst suggesting again the capacity of the catalyst to accelerate the degradation of PBS in these conditions.

The degradation is more gradual in water than in methanol. Both conditions showed a clear shift of the SEC trace toward lower molar mass but in methanol, low molecular weight shoulder peaks were visible, indicating a nonuniform degradation (Figure 2C,D). Alongside the increase of mass loss, this indicates that degradation was faster at the surface of the film. In deionized water, it seems that the water had more time to diffuse into the film resulting in more bulk degradation [79, 80].

### 2.4 Degradation of PBAT Films in Methanol

**FIGURE 3:**
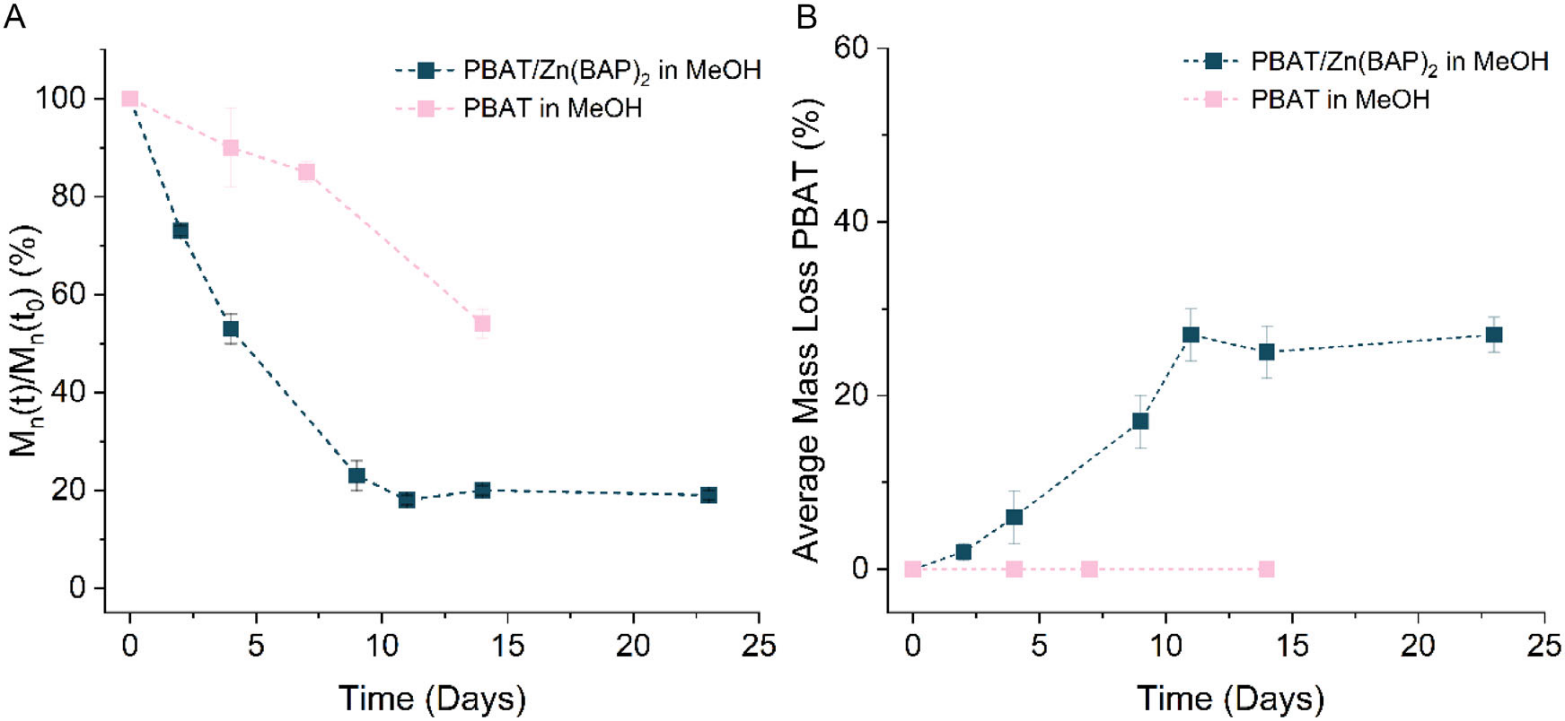
(A) Average % mass loss of PBAT after incubation in methanol at 50°C. (B) Average molar mass of PBAT relative to average initial molar mass over time in methanol at 50°C.

To assess the versatility of the catalyst, embedded films of PBAT were also incubated in methanol at 50°C. A slow increase in mass loss was observed for catalyst‐embedded films with a clear shift toward lower molar mass, reaching an average of 18 ± 1% of the initial molar mass after 11 days (Figure 3). The films with no catalyst reached an average of 54 ± 4% of the initial molar mass after 14 days. This highlights the efficiency of the catalyst in accelerating the degradation of PBAT as well as PBS. A mass loss of around 30% after 11 days compares favorably to the dizinc complex from Wu et al., which afforded a 20% yield of 1,4‐BDO after 14 days in water [75]. The increase in mass loss indicates some surface degradation with SEC traces broadening over time, but the decrease in molar mass indicates mainly bulk degradation, in keeping with the observations of Wu [75]. The slower degradation of PBAT films containing catalyst compared to PBS is probably explained by the aromatic content of PBAT. Sander and coworkers demonstrated that increasing terephthalate content significantly reduced biodegradation in soil, which was attributed to the formation of crystalline domains of terephthalate and butylene units [81]. It is likely that this effect accounts for the reduced rate of PBAT degradation in methanol compared to PBS.

### 2.5 Stability of Embedded Films

**FIGURE 4:**
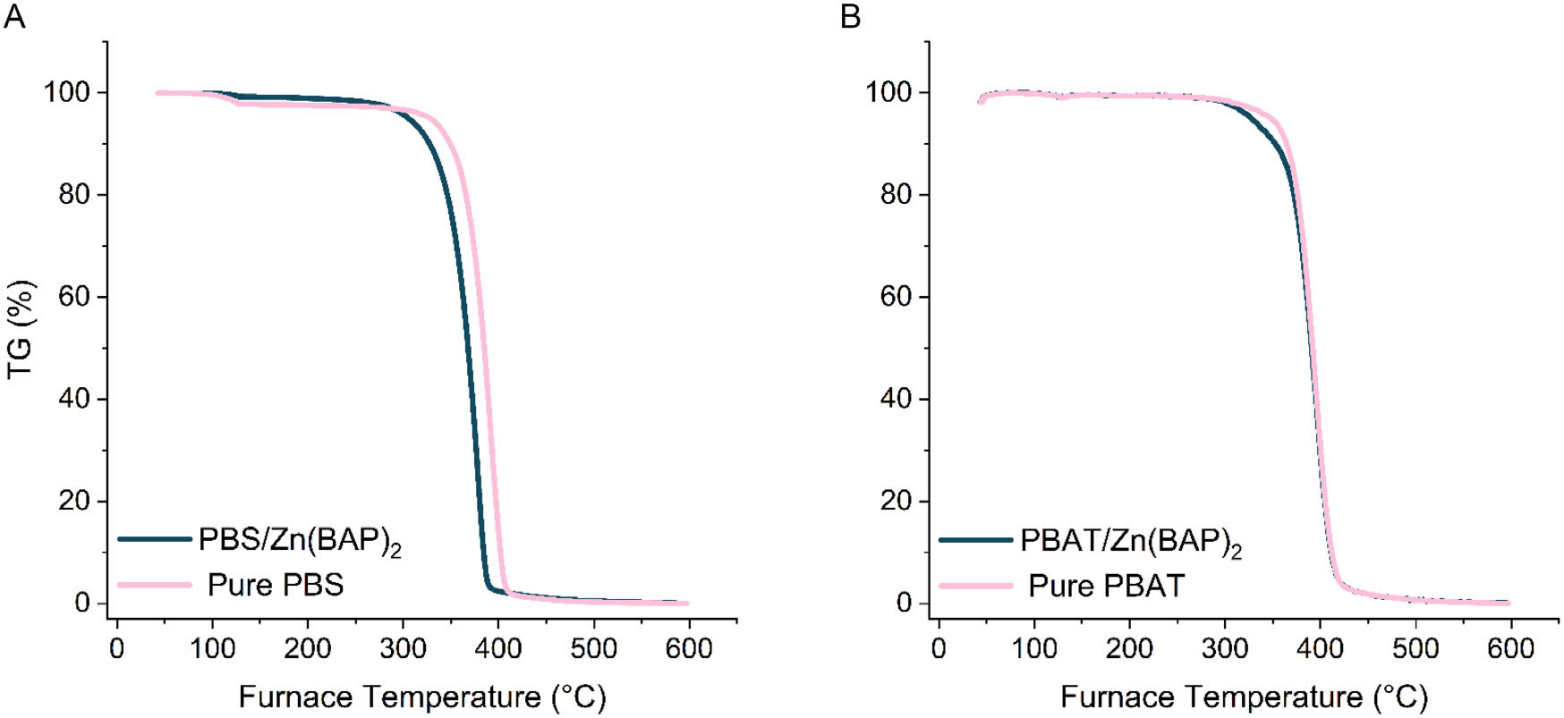
Thermal stability of (A) pure PBS and PBS/Zn(BAP)_2_ (B) pure PBAT and PBAT/Zn(BAP)_2_.

The thermal stability of the Zn(BAP)_2_‐embedded polymer films was evaluated and compared to catalyst‐free samples (Figure 4). The temperature at 5% degradation (*T*_d,5%_) for PBS decreased from 342.9 to 315.6°C when catalyst was incorporated alongside a decrease in the inflection point (*T*_d_, inflection) from 392.5 to 377.9°C. Similarly, the *T*_d,5%_ for PBAT decreased from 352.4 to 333.6°C with catalyst present. However, a slight increase in *T*_d_, inflection was observed from 395.5 to 398.3°C with catalyst. This indicates that Zn(BAP)_2_ incorporation marginally decreases the temperature of thermal degradation for PBS and PBAT. These polymers are typically used for low‐temperature applications such as packaging, carrier bags, and mulch films, and the slight decrease in thermal stability is highly unlikely to have a negative effect on their utility. The mechanical properties of the degraded polymers were not tested due to the brittleness and fragility of the collected material. This can be seen from the postdegradation images shown in Figure S7.

Another aspect of the stability of embedded films is the extent to which the catalyst reacts in the solid phase within the polymer matrix or if dissolved catalyst is the primary degradation agent. For the degradation in water, catalyst leaching is unlikely due to the insolubility of Zn(BAP)_2_ in water (Figure S8). Niu and coworkers concluded that adding a dizinc complex to PBAT did not affect biocompatibility and that solid‐phase catalyst was the active component. However, specific testing of our system would be required to assess these parameters. Furthermore, the extent of catalyst leaching in methanol requires further investigation.

## 3 Conclusion

The degradation of PBS and PBAT was investigated through zinc‐catalysed methanolysis. The conditions in solution were varied and showed that methanol concentration, catalyst loading, and temperature all had a positive correlation with PBS conversion and DMS yield. When the reaction was performed in neat methanol, full conversion was reached after 1 h with 85% DMS yield. PBAT was shown to readily depolymerise under the same conditions to give high product yields. Embedding the catalyst into the polymers and incubating with methanol or water resulted in significant mass loss and a reduction in molar mass when compared with the pure polymer controls. The degradation was quicker in methanol than in water, which could be a result of catalyst deactivation. Furthermore, PBS degradation was significantly quicker than PBAT degradation as would be expected from an aromatic polyester. This work demonstrates the potential for efficient monomer recovery through chemical recycling of PBAT and PBS as well as improving degradability through the incorporation of an active catalyst.

## Supporting Information

Additional supporting information can be found online in the Supporting Information section. **Supporting Fig. S1:** ^1^H NMR spectrum (400 MHz, CDCl_3_) of BAP ligand. **Supporting Fig. S2:** ^1^H NMR spectrum (400 MHz, CDCl_3_) of Zn(BAP)_2_. **Supporting Fig. S3:** ^1^H NMR spectrum (400 MHz, CDCl_3_) of PBS. **Supporting Fig. S4:** ^1^H NMR spectrum (400 MHz, CDCl_3_) of PBS methanolysis (Table S3, entry 1) showing O‐CH2‐O peaks of succinate subunits. Line fitting has been used to calculate the area of the three peaks. **Supporting Fig. S5:** ^1^H NMR spectrum (400 MHz, CDCl_3_) of PBAT. **Supporting Fig. S6:** ^1^H NMR spectrum (400 MHz, CDCl_3_) of PBAT depolymerisation with highlighted signals used to calculate conversion of PBA and PBT subunits. **Supporting Fig. S7:** ^1^H NMR spectrum (400 MHz, CDCl_3_) of PBAT depolymerisation showing line fitting region of aromatic terephthalate protons (δ = 8.103 – 8.112 ppm). **Supporting Fig. S8:** Example images of degraded PLA/Zn(BAP)_2_ films. **Supporting Fig. S9:** ^1^H NMR spectrum (500 MHz, D_2_O) of Zn(BAP)_2_. **Supporting Table S1:** Catalyst loading comparison. 80°C, 10 eq. MeOH, Zn(BAP)_2_, 48 hours. **Supporting Table S2:** Temperature comparison. 10 eq. MeOH, 4 wt% Zn(BAP)_2_, 48 hours. **Supporting Table S3:** Methanol loading comparison. 80 °C 4 wt% Zn(BAP)_2_, 48 hours. **Supporting Scheme S1:** Synthesis of BAP ligand. **Supporting Scheme S2:** Synthesis of Zn(BAP)_2_.

## Funding

This work was supported by NERC (E/V007246/1); Engineering and Physical Sciences Research Council (EP/R027128/1); Royal Society (URF\R\221027, UF/16002).

## Conflicts of Interest

The authors declare no conflicts of interest.

## Supporting information

Supplementary Material

## Data Availability

The data that support the findings of this study are available in the supplementary material of this article.
