# Supplementary material for: Zinc‐Catalysed Depolymerization of Poly(Butylene Succinate) and Poly(Butylene Adipate‐co‐Terephthalate) and Enhanced Degradation of Catalyst‐Polymer Composite Films
Source: ChemSusChem. 2025 Dec 1;19(1):e202502332. doi: 10.1002/cssc.202502332 (PMC12767557; doi:10.1002/cssc.202502332)
Supplement: Supplementary file 1 — Supplementary Material [file CSSC-19-e202502332-s001.pdf]

Supporting Information

Zinc-catalysed depolymerisation of poly(butylene succinate) and poly(butylene adipate-*co*-terephthalate) and enhanced degradation of catalyst-polymer composite films

Fannie Burgevin,[a†] Jack A. Stewart,[a†] Annie May,[a] Matthew J. Cullen,[a,b] Antoine Buchard,[c] Matthew G. Davidson[a,b] and Matthew D. Jones[a,b*]

[a] Department of Chemistry, University of Bath, Claverton Down, Bath, United Kingdom, BA2 7AY. E-mail: mj205@bath.ac.uk
[b] Institute of Sustainability and Climate Change, University of Bath, Claverton Down, Bath, United Kingdom, BA2 7AY
[c] Department of Chemistry, Green Chemistry Centre of Excellence, University of York, York, United Kingdom, YO10 5DD
† These two authors contributed equally to this work

Table of Contents

1. General Considerations and Procedures 2

1.1 Chemicals and equipment 2

2. Synthesis and Characterisation of $Zn(BAP)_2$ 3

2.1 Synthesis and characterisation of BAP ligand 3
2.2 Catalyst Synthesis and Characterisation 4

3. Polymer Characterisation 6

3.1 PBS characterisation and depolymerisation 6
3.2 PBAT characterisation and depolymerisation 8

4. PBS Methanolysis 10

4.1 Optimisation of PBS methanolysis in THF 10

5. Composite film Degradation Data 11

5.1 PBS and PBS/$Zn(BAP)_2$ in methanol 11
5.2 PBS and PBS/$Zn(BAP)_2$ in water 12
5.3 PBAT and PBAT/$Zn(BAP)_2$ in methanol 13
5.4 Images of degraded films 14

6. $Zn(BAP)_2$ solubility in water 14

1. General Considerations and Procedures

1.1 Chemicals and equipment

All chemicals were obtained commercially from Sigma-Aldrich and used as received. Ligand was prepared using standard literature procedures in air. $Zn(BAP)_2$ was synthesised under an inert atmosphere (argon) using standard Schlenk line techniques, dry solvents, and oven-dried glassware. Complex was stored in a vial at ambient conditions.

^{1}H and $^{13}C\{^{1}H\}$ NMR spectra of ligands, complexes, polymerisations, and degradations were obtained on a Bruker 400 MHz or 500 MHz instrument. Coupling constants (*J*) are provided in Hertz (Hz) and the following abbreviations were used to report the spectra: (s) singlet, (d) doublet, (dd) doublet of doublets, (t) triplet, (p) pentet, (m) multiplet. Characterisation of ligands and metal complexes used C_6D_6 or $CDCl_3$ as the NMR solvent. Quantification of depolymerisations used $CDCl_3$ as the NMR solvent.

SEC was performed using chloroform as the eluent. Samples were dissolved in chloroform to give a solution of 2 mg/mL. Before injection, analyte samples were filtered through a PTFE membrane with 0.45 µm pore size. An Agilent infinity II equipped with differential refractive index (DRI) was used. The system was equipped with 2 x PLgel mixed C columns (300 x 7.5 mm) and a PLgel guard column. Samples were run at 1 mL/min at 40 °C. Polystyrene standards were used for the calibration between 195,500 and 370 $gmol^{-1}$.

TGA was carried out using A Setsys Evolution TGA 16/18 from Setaram; Calisto was used to process data. The samples were loaded into a 170 µL alumina crucible and the analytical chamber purged with argon (200 mL/min) for 20 minutes prior to analysis. The samples were heated under an argon flow (20 mL/min) from 30 °C to 600 °C at a rate of 10K/min.

1.2 General procedures

PBS depolymerisation in THF: PBS (0.25 g, 1.5 mmol) was added to an oven dried J. Young's flask under an argon atmosphere. Catalyst (2 – 8 wt%, 5 – 20 mg, 0.4 – 1.7 mol% relative to polymer repeat unit) was added to the flask which was then submerged in an oil bath set to the required temperature. THF (4 mL) was added to dissolve the PBS and, when dissolved, methanol (5 – 50 equiv. with respect to polymer repeat unit, 0.3 – 3.0 mL) was added. After the desired amount of time, ^{1}H NMR spectroscopy was used to analyse the final reaction mixture.

PBS/PBAT depolymerisation in methanol: PBS or PBAT (0.25 g) was added to an oven dried J. Young's flask under an argon atmosphere. Catalyst (2 – 8 wt%, 5 – 20 mg, 0 – 3.9 mol% relative to polymer repeat unit) and methanol (33 equiv. relative to polymer repeat unit, 2 mL) was added to the flask which was then submerged in an oil bath set to the required temperature. After the desired amount of time, ^{1}H NMR spectroscopy was used to analyse the final reaction mixture.

Polymer-catalyst composite thin film preparation: Polymer pellets (1.00 g) and $Zn(BAP)_2$ (4 wt%, 0.04 g, 0.065 mmol) were stirred and dissolved in a suitable solvent and poured into a PTFE dish. The solvent was left to evaporate, leaving a white solid. The solid was hot-pressed at 120 °C to produce a film. The same process was followed without catalyst for the blank runs with pure polymer.

Degradation of thin films: A portion of the film (1cm x 1cm) was weighed and immersed into a vial of methanol or DI water, which was left in an incubator at 50 °C. After the required amount of time the

film was removed from the incubator, rinsed with deionised water, and left to dry. Once dry, the films were weighed. Each timepoint was repeated in triplicate to ensure accurate results.

2. Synthesis and Characterisation of $Zn(BAP)_2$

2.1 Synthesis and characterisation of BAP ligand

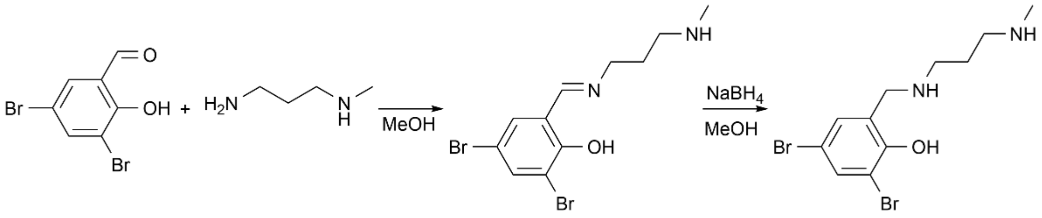

Scheme S1: Synthesis of BAP ligand.

A literature procedure was followed for ligand and complex synthesis.[1] 3,5-Dibromosalicyl aldehyde (2.80 g, 10 mmol) was dissolved in methanol (25 mL). 3-Dimethylamino-1-propylamine (1.25 mL, 10 mmol) was then added dropwise and the resulting reaction mixture was stirred for three hours. Rapid precipitation of a white powder was observed upon addition of sodium borohydride (0.96 g, 25 mmol) to the reaction mixture. The mixture was stirred for one hour, followed by addition of deionised water (25 mL) to quench the reaction. The solvent was removed *in vacuo* to give a white powder, which was washed with deionised water (20 mL) (2.97 g, 81 %).

^{1}H NMR (400 MHz, $CDCl_3$) δ 7.52 (d, J = 2.4 Hz, 1H, H_A), δ 7.04 (d, J = 2.8 Hz, 1H, H_B) δ 3.95 (s, 2H, H_C) δ 2.72 (t, J = 6.4 Hz, 2H, $H_{D/}H_E$) δ 2.34 (t, J = 6.4 Hz, 2H, $H_{D/}H_E$) δ 2.20 (s, 3H, H_G) δ 1.69 (p, J = 6.4 Hz, 2H, H_F).

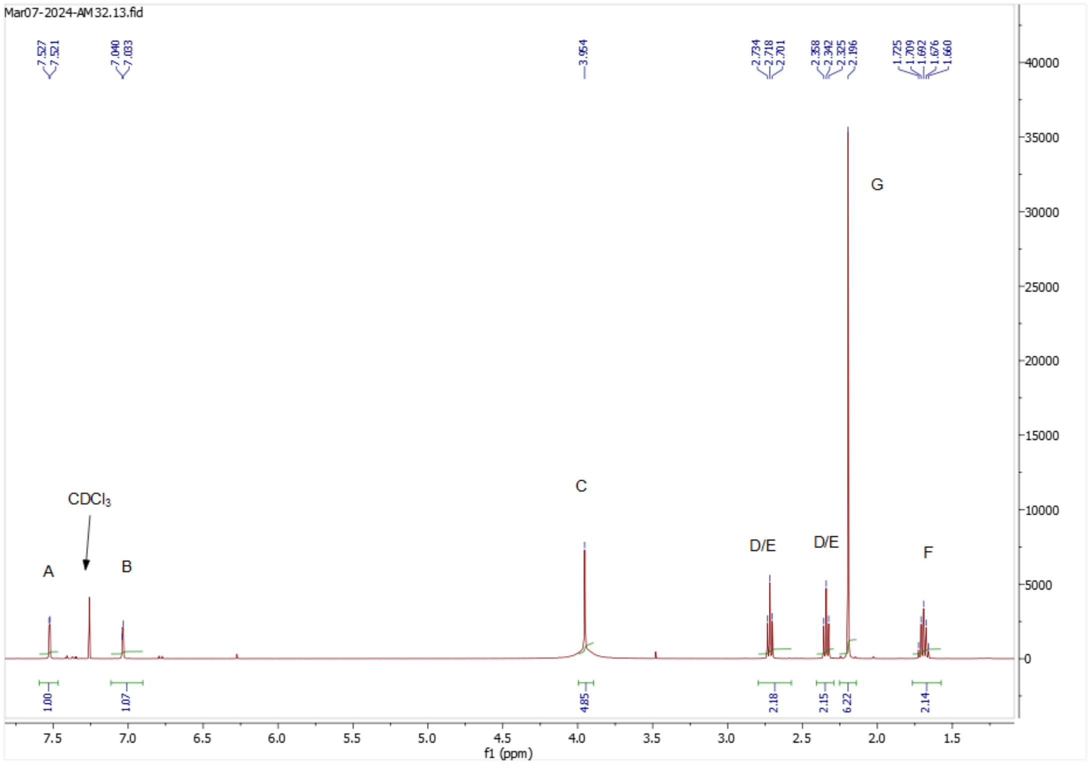

Figure S1: ^{1}H NMR spectrum (400 MHz, $CDCl_3$) of BAP ligand.

2.2 Catalyst Synthesis and Characterisation

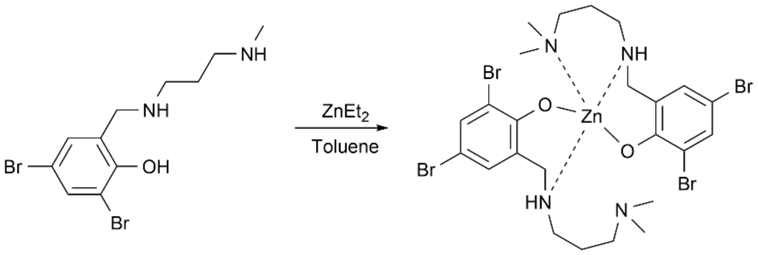

Scheme S2: Synthesis of Zn(BAP)$_2$.

Ligand (1.46 g, 4.0 mmol) was added to a Schlenk flask and dried under vacuum. Dry toluene (20 mL) was added under an argon atmosphere. Diethylzinc (2 mL, 20 mmol) was added dropwise to the reaction flask and smoke was observed. The solution was stirred at 80 °C for 48 hours. The resulting white solid was isolated by decanting the liquid under an argon atmosphere and dried *in vacuo* (0.78 g, 49 %).

^{1}H NMR (400 MHz, $CDCl_3$) δ 7.52 (d, J = 2.4 Hz, 1H, H_A), δ 7.02 (d, J = 2.8 Hz, 1H, H_B) δ 3.85 (br s, 2H, H_C) δ 3.03 (br s, 2H, $H_{D/}H_E$) δ 2.46 (s, 2H, $H_{D/}H_E$) δ 2.24 (s, 6H, H_G) δ 1.78 (br s, 2H, H_F).

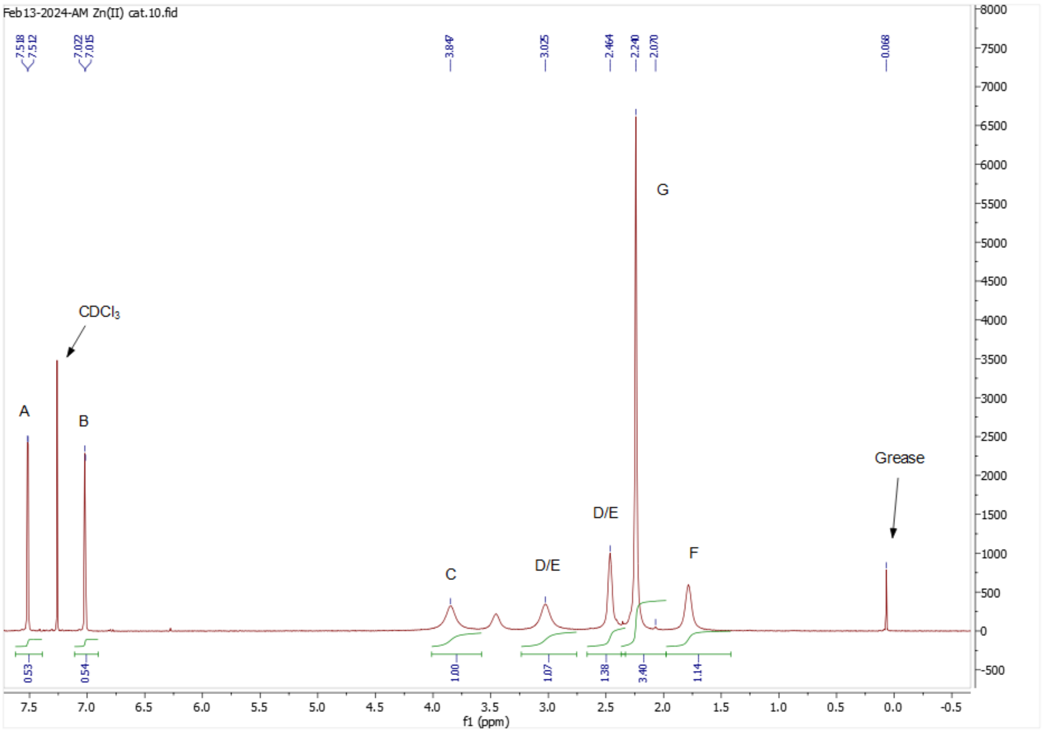

Figure S2: ^{1}H NMR spectrum (400 MHz, $CDCl_3$) of $Zn(BAP)_2$.

3. Polymer Characterisation

3.1 PBS characterisation and depolymerisation

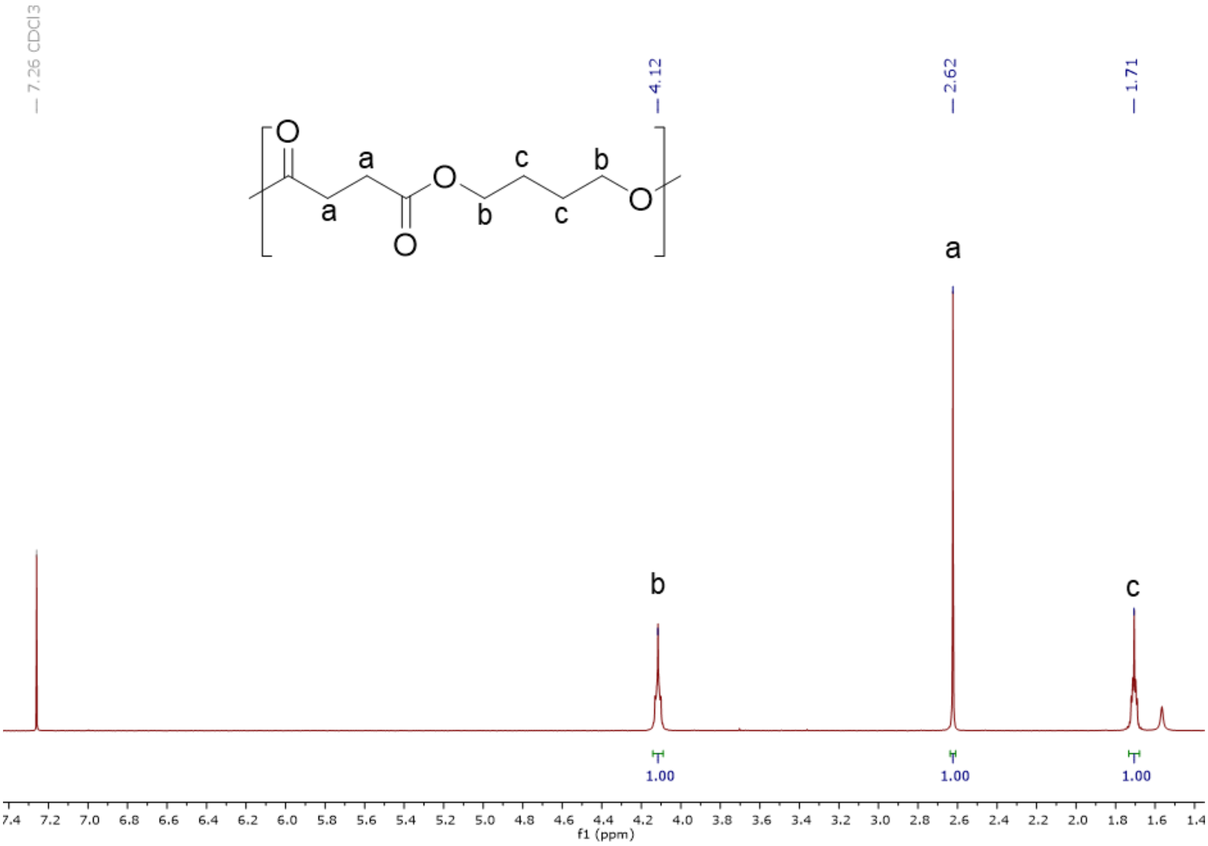

Figure S3: ^{1}H NMR spectrum (400 MHz, $CDCl_3$) of PBS.

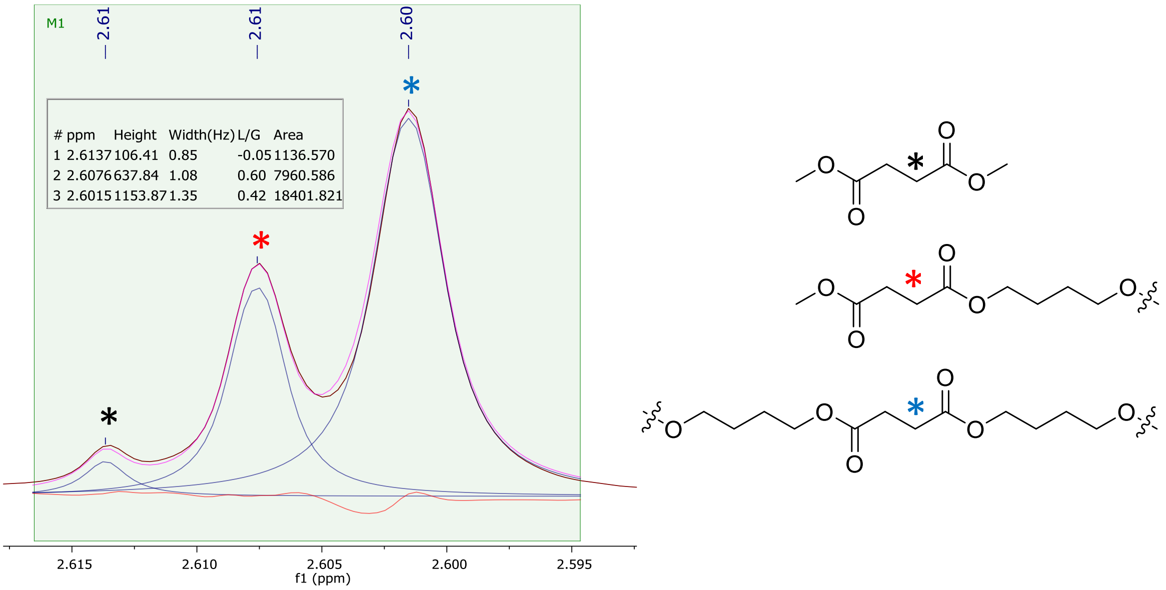

Figure S4: ^{1}H NMR spectrum (400 MHz, $CDCl_3$) of PBS methanolysis (**Table S3**, **entry 1**) showing O-CH_2-O peaks of succinate subunits. Line fitting has been used to calculate the area of the three peaks.

The peak areas from **Figure S4** can be used to caluclate the percentage of each succinate environment:

$$[DMS] = \frac{*}{*.*.*} \times 100 = \frac{1136.570}{1136.570 + 7960.586 + 18401.821} \times 100 = 4\%$$

$$[Chain\ End] = \frac{*}{*.*.*} \times 100 = \frac{7960.586}{1136.570 + 7960.586 + 18401.821} \times 100 = 29\%$$

$$[PBS] = \frac{*}{*.*.*} \times 100 = \frac{18401.821}{1136.570 + 7960.586 + 18401.821} \times 100 = 67\%$$

Using the relative quantities calculate above, it is possible to calculate the following three parameters. X_{PBS} represents the conversion of PBS to all products. S_{DMS} and Y_{DMS} represent the selectivity and yield of DMS.

$$X_{PBS} = \left(1 - \frac{[PBS]}{[PBS]_0}\right) \times 100 = \left(1 - \frac{67}{100}\right) \times 100 = 33\%$$

$$S_{DMS} = \left(\frac{[DMS]}{[PBS]_0 - [PBS]}\right) \times 100 = \left(\frac{4}{100 - 67}\right) \times 100 = 12\%$$

$$Y_{DMS} = \frac{X_{PBS} S_{DMS}}{100} Y_{PBS} = \frac{33 \times 12}{100} = 4\%$$

3.2 PBAT characterisation and depolymerisation

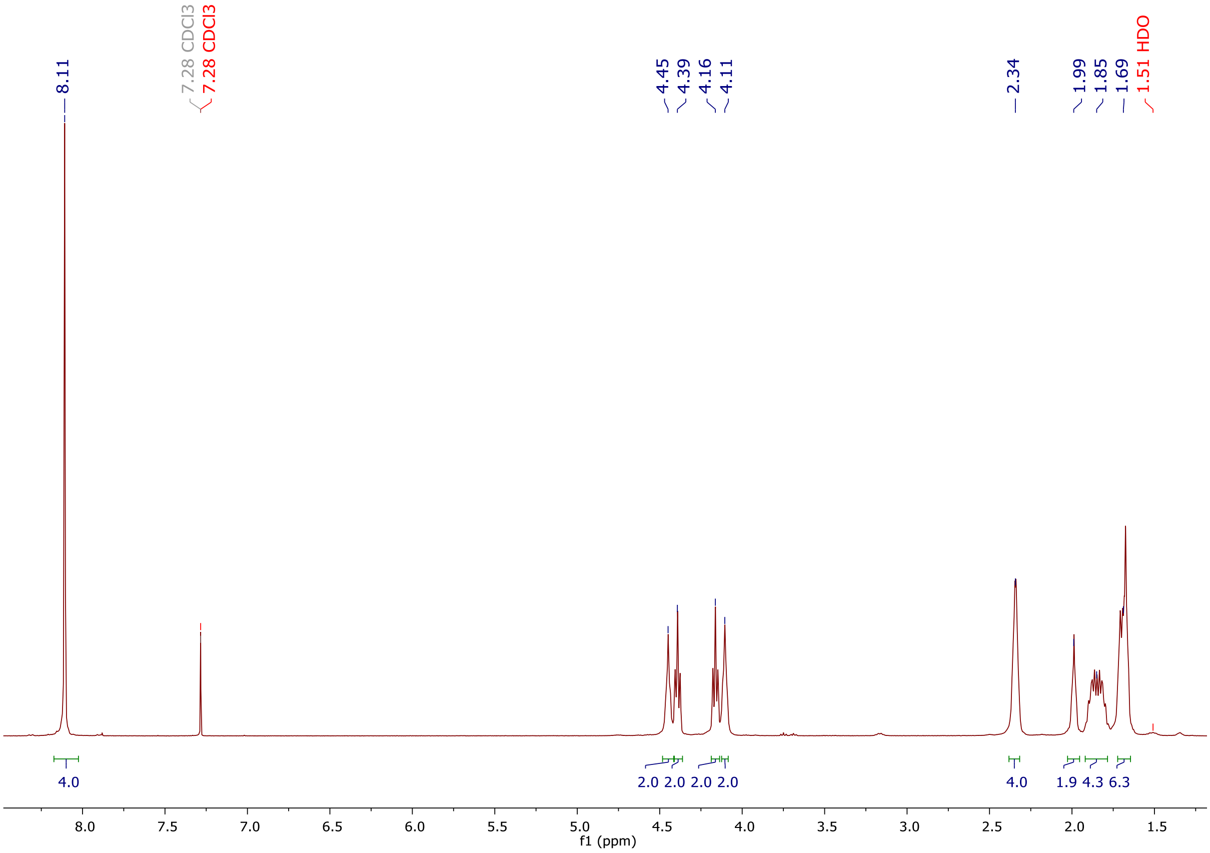

Figure S5: ^1H NMR spectrum (400 MHz, $CDCl_3$) of PBAT.

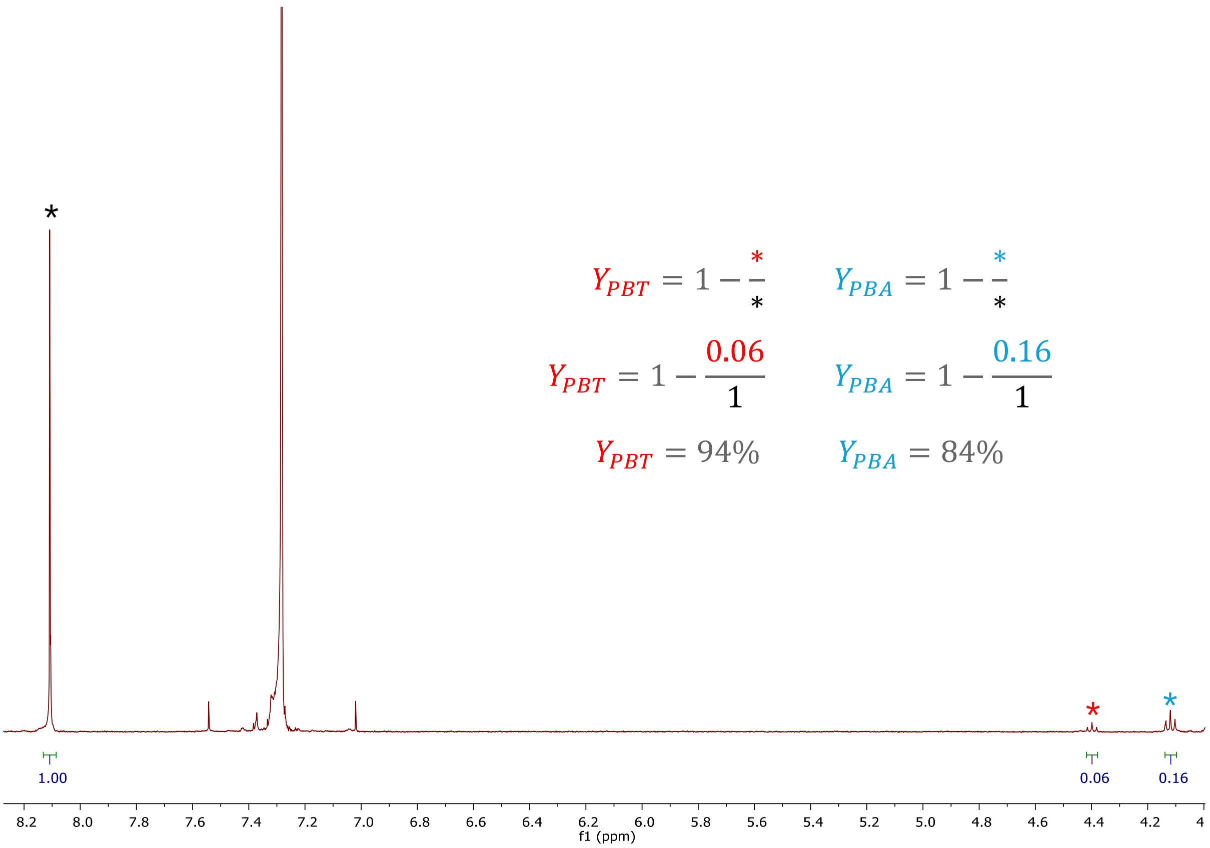

Figure S6: ^{1}H NMR spectrum (400 MHz, $CDCl_3$) of PBAT depolymerisation with highlighted signals used to calculate cponversion of PBA and PBT subunits.

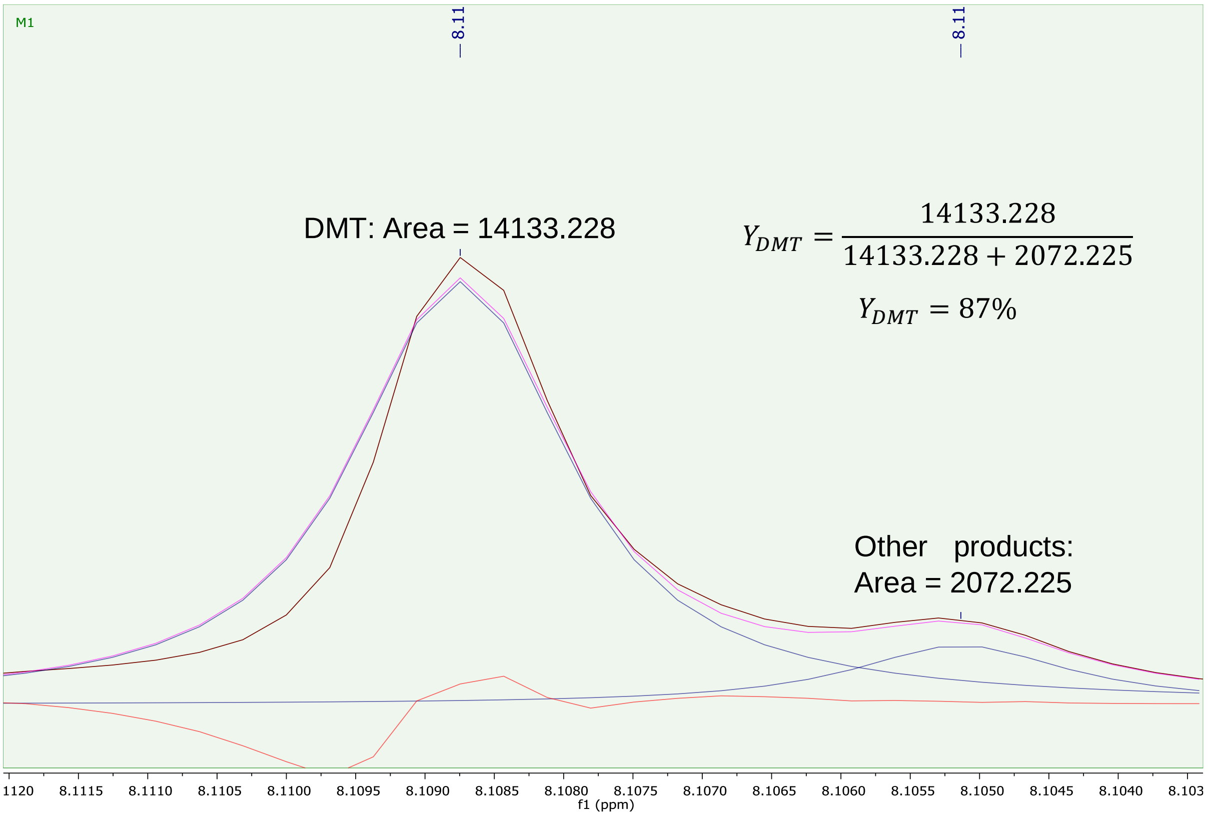

Figure S6: ^{1}H NMR spectrum (400 MHz, $CDCl_3$) of PBAT depolymerisation showing line fitting region of aromatic terephthalate protons (δ = 8.103 – 8.112 ppm).

4. PBS Methanolysis

4.1 Optimisation of PBS methanolysis in THF

Table S1 Catalyst loading comparison. 80°C, 10 eq. MeOH, Zn(BAP)$_2$, 48 hours.[a]

Entry	Loading [wt%]	*X* [%][b]	*S* [%][b]	*Y* [%][b]
1	2	33	13	4
2	4	84	47	40
3	6	87	43	37
4	8	95	63	60

[a] Catalyst loading comparison. Conditions: 2 – 8 wt% Zn(BAP)$_2$ (5 – 20 mg, 0.4 – 1.7 mol% relative to polymer repeat unit), 10 equiv. MeOH relative to polymer repeat unit (0.6 mL), 4 mL THF, 80 °C, 48 h. [b] Calculated from ^{1}H NMR spectroscopy.

Table S2 Temperature comparison. 10 eq. MeOH, 4 wt% Zn(BAP)$_2$, 48 hours.[a]

Entry	Temp [°C]	*X* [%][b]	*S* [%][b]	*Y* [%][b]
1	30	5	0	0
2	50	9	1	0
3	80	84	47	40
4	100	98	63	62

[a] Temperature comparison. Conditions: 4 wt% Zn(BAP)$_2$ (10 mg, 0.9 mol% relative to polymer repeat unit), 10 equiv. MeOH relative to polymer repeat unit (0.6 mL), 4 mL THF, 30 – 100 °C, 48 h. [b] Calculated from ^{1}H NMR spectroscopy.

Table S3 Methanol loading comparison. 80 °C 4 wt% Zn(BAP)$_2$, 48 hours.[a]

Entry	MeOH Loading [eq.]	*X* [%][b]	*S* [%][b]	*Y* [%][b]
1	5	33	12	4
2	10	84	47	40
3	20	95	62	59
4	50	96	71	68

[a] Methanol loading comparison. Conditions: 4 wt% Zn(BAP)$_2$ (10 mg, 0.9 mol% relative to polymer repeat unit), 5 – 50 equiv. MeOH relative to polymer repeat unit (0.3 – 3.0 mL), 4 mL THF, 80 °C, 48 h. [b] Calculated from ^{1}H NMR spectroscopy.

5. Composite film Degradation Data

5.1 PBS and PBS/Zn(BAP)$_2$ in methanol

Time / hours		Mass before / g	boat / g	boat + film / g	mass after / g	mass change / g	% mass loss	Average mass loss	Std Dev	Mn	Average Mn	%	Average %	Std Dev	Dispersity	Average Dispersity
										27902					2.604903	
										27074	25945				2.700524	2.821922
										22858					3.160338	
		0.044	0.602	0.641	0.039	0.005	11%			7387		28%			2.385136	
6	0.25	0.043	0.656	0.695	0.039	0.004	9%	9%	2%	6691	6780	26%	26%	2%	2.072485	2.148016
		0.04	0.659	0.696	0.037	0.003	8%			6262		24%			1.986426	
		0.04	0.58	0.61	0.03	0.01	25%			4215		16%			1.665006	
24	1	0.044	0.699	0.733	0.034	0.01	23%	25%	2%	4036	4084	16%	16%	0%	1.666749	1.675636
		0.039	0.677	0.705	0.028	0.011	28%			4002		15%			1.695152	
		0.046	0.669	0.702	0.033	0.013	28%			3942		15%			1.570269	
48	2	0.037	0.667	0.695	0.028	0.009	24%	28%	2%	3877	3822	15%	15%	0%	1.615940	1.573491
		0.04	0.679	0.707	0.028	0.012	30%			3648		14%			1.534265	
		0.042	0.678	0.706	0.028	0.014	33%			3670		14%			1.561580	
72	3	0.041	0.692	0.719	0.027	0.014	34%	33%	1%	3680	3646	14%	14%	0%	1.551630	1.539130
		0.039	0.692	0.719	0.027	0.012	31%			3588		14%			1.504181	
		0.036	0.69	0.714	0.024	0.012	33%			3660		14%			1.532240	
96	4	0.04	0.695	0.725	0.03	0.01	25%	31%	5%	3395	3443	13%	13%	1%	1.567894	1.511488
		0.048	0.716	0.747	0.031	0.017	35%			3274		13%			1.434331	
		0.048	0.678	0.709	0.031	0.017	35%			3399		13%			1.480730	
168	7	0.052	0.692	0.725	0.033	0.019	37%	37%	2%	3280	3392	13%	13%	0%	1.472866	1.470516
		0.042	0.692	0.717	0.025	0.017	40%			3496		13%			1.457952	

Time / hours		Mass before / g	boat / g	boat + film / g	mass after / g	mass change / g	% mass loss	Average mass loss	Std Dev	Mn	Average Mn	%	Average %	Std Dev	Dispersity	Average Dispersity
										51569						
										43629	49887					
										54464						
		0.07	0.658	0.726	0.068	0.0020	3%			49398		99%			2.791509778	
48		0.053	0.654	0.707	0.053	0.0000	0%	1%	1%	47862	49411	96%	99%	3%	2.959425014	2.828126
		0.06	0.691	0.751	0.06	0.0000	0%			50972		102%			2.73344189	
		0.06	0.697	0.741	0.044	0.0160	27%			21536		43%			2.159314636	
168		0.057	0.652	0.694	0.042	0.0150	26%	4%	10%	19801	21283	40%	43%	2%	2.191151962	2.252394
		0.046	0.697	0.741	0.044	0.0020	4%			22512		45%			2.406716418	
		0.06	0.669	0.728	0.059	0.0010	2%			23748		48%			3.729703554	
2 weeks		0.064	0.671	0.732	0.061	0.0030	5%	3%	1%	25685	25245	51%	51%	2%	3.500447732	3.556726
		0.061	0.659	0.719	0.06	0.0010	2%			26303		53%			3.440025853	

5.2 PBS and PBS/Zn(BAP)$_2$ in water

Water

Sample	Time / hours		Mass before / g	boat / g	boat + film / g	mass after / g	mass change / g	% mass loss	Average mass loss	Std Dev	Mn	Average Mn	%	Average %	Std Dev	Dispersity	Average Dispersity
PBS-ref 1											27902					2.604903	
PBS-ref 2											27074	25945				2.700524	2.821922
PBS-ref 3											22858					3.160338	
PBS-T1 (1)			0.039	0.698	0.738	0.04	-0.001	-3%			24051		93%			2.517401	
PBS-T1 (2)	6	0.25	0.044	0.632	0.674	0.042	0.002	5%	5%	4%	19003	21581	73%	83%	8%	3.237436	2.853927
PBS-T1 (3)			0.049	0.623	0.659	0.036	0.013	27%			21688		84%			2.806944	
PBS-T2 (1)			0.04	0.707	0.747	0.04	0	0%			18107		70%			2.599989	
PBS-T2 (2)	24	1	0.042	0.672	0.714	0.042	7.63278E-17	0%	2%	2%	16120	17685	62%	68%	4%	3.092246	2.782530
PBS-T2 (3)			0.044	0.707	0.749	0.042	0.002	5%			18828		73%			2.655354	
PBS-T3 (1)			0.047	0.664	0.71	0.046	0.001	2%			16311		63%			2.523021	
PBS-T3 (2)	48	2	0.039	0.653	0.693	0.04	-0.001	-3%	1%	2%	17521	17361	68%	67%	3%	2.589864	2.523209
PBS-T3 (3)			0.037	0.665	0.702	0.037	7.63278E-17	0%			18251		70%			2.456742	
PBS-T4 (1)			0.04	0.667	0.705	0.038	0.002	5%			19811		76%			2.47403	
PBS-T4 (2)	96	4	0.04	0.662	0.702	0.04	7.63278E-17	0%	4%	3%	16985	18226	65%	70%	5%	2.483014	2.436731
PBS-T4 (3)			0.044	0.745	0.786	0.041	0.003	7%			17882		69%			2.353148	
PBS-T5 (1)			0.044	0.664	0.708	0.044	6.93889E-17	0%			16541		64%			2.315156	
PBS-T5 (2)	168	7	0.047	0.653	0.699	0.046	0.001	2%	1%	3%	15219	15793	59%	61%	2%	2.433406	2.415280
PBS-T5 (3)			0.045	0.665	0.712	0.047	-0.002	-4%			15619		60%			2.497279	
PBS-T6(1)			0.053	0.572	0.624	0.052	0.001	2%			14369		55%			2.427378	
PBS-T6(2)	216	9	0.04	0.642	0.68	0.038	0.002	5%	4%	1%	13527	13236	52%	51%	4%	2.36867	2.503291
PBS-T6(3)			0.046	0.649	0.693	0.044	0.002	4%			11811		46%			2.713826	
PBS-T7(1)			0.036	0.665	0.7	0.035	0.001	3%			12814		49%			2.3218355	
PBS-T7(2)	408	17	0.051	0.647	0.697	0.05	0.001	2%	3%	1%	12310	12603	47%	49%	1%	2.4948822	2.377832
PBS-T7(3)			0.046	0.662	0.706	0.044	0.002	4%			12684		49%			2.316777	
PBS-T8(1)			0.047	0.66	0.706	0.046	0.001	2%			12456		48%			2.2812299	
PBS-T8(2)	504	21	0.045	0.607	0.651	0.044	0.001	2%	1%	1%	11683	12203	45%	47%	1%	2.4217239	2.360755
PBS-T8(3)			0.046	0.617	0.663	0.046	0	0%			12470		48%			2.3793103	

BLANK	Time / hours		Mass before / g	boat / g	boat + film / g	mass after / g	mass change / g	% mass loss	Average mass loss	Std Dev	Mn	Average Mn	%	Average %	Std Dev	Dispersity	Average Dispersity
PBS -ref 1											51569						
PBS -ref 2											43629	**49887**					
PBS -ref 3											54464						
BLANK-PBS-T1 (1)			0.045	0.674	0.719	0.045	0.0000	0%			50563		101%			2.8384985	
BLANK-PBS-T1 (2)	48		0.053	0.686	0.737	0.051	0.0020	4%	2%	2%	52924	**51239**	106%	103%	2%	2.7084687	2.794141384
BLANK-PBS-T1 (3)			0.043	0.655	0.697	0.042	0.0010	2%			50230		101%			2.8354569	
BLANK-PBS-T2 (1)			0.06	0.669	0.73	0.061	-0.0010	-2%			54309		109%			2.554862	
BLANK-PBS-T2 (2)	168 (1 week)		0.064	0.66	0.723	0.063	0.0010	2%	0%	1%	54606	**53017**	109%	106%	4%	2.5445189	2.623426495
BLANK-PBS-T2 (3)			0.04	0.64	0.68	0.04	0.0000	0%			50135		100%			2.7708986	
BLANK-PBS-T3 (1)			0.041	0.684	0.724	0.04	0.0010	2%			49270		99%			2.6122793	
BLANK-PBS-T3 (2)	3 weeks		0.04	0.708	0.747	0.039	0.0010	2%	1%	2%	48761	**46536**	98%	93%	7%	2.5346076	2.713600537
BLANK-PBS-T3 (3)			0.049	0.631	0.681	0.05	-0.0010	-2%			41576		83%			2.9939148	

5.3 PBAT and PBAT/Zn(BAP)$_2$ in methanol

MeOH Sample	Time / hours	Mass before / g	boat / g	boat + film / g	mass after / g	mass change / g	% mass loss	Average mass loss	Std Dev	Mn	Average Mn	%	Average %	std dev
PBAT-ref 1										49799	49799			
PBAT-ref 2														
PBAT-ref 3														
PBAT-T1 (1)	2	0.044	0.661	0.704	0.043	0.001	2%	2%	1%	35823	36268	72%	73%	1%
PBAT-T1 (2)		0.043	0.684	0.726	0.042	0.001	2%			37223		75%		
PBAT-T1 (3)		0.044	0.66	0.704	0.044	6.93889E-17	0%			35757		72%		
PBAT-T2 (1)	96	0.05	-	-	0.047	0.003	6%	6%	3%	25931	26489	52%	53%	3%
PBAT-T2 (2)		0.047	-	-	0.043	0.004	9%			28400		57%		
PBAT-T2 (3)		0.046	-	-	0.045	0.001	2%			25136		50%		
PBAT-T3 (1)	9 days	0.045	0.669	0.708	0.039	0.006	13%	17%	3%	11944	11599	24%	23%	3%
PBAT-T3 (2)		0.047	0.593	0.63	0.037	0.01	21%			9728		20%		
PBAT-T3 (3)		0.051	0.707	0.75	0.043	0.008	16%			13126		26%		
PBAT-T4 (1)	11 days - Friday 22nd	0.049	0.626	0.662	0.036	0.013	27%	27%	3%	9610	9081	19%	18%	1%
PBAT-T4 (2)		0.048	0.605	0.638	0.033	0.015	31%			8973		18%		
PBAT-T4 (3)		0.042	0.616	0.648	0.032	0.01	24%			8659		17%		
PBAT-T5 (1)	2 weeks - Monday 25th	0.047	0.667	0.702	0.035	0.012	26%	25%	3%	9459	9808	19%	20%	1%
PBAT-T5 (2)		0.051	0.628	0.667	0.039	0.012	24%			10237		21%		
PBAT-T5 (3)		0.046	0.706	0.744	0.038	0.008	17%			9728		20%		
PBAT-T6 (1)	23 days - Wednesday 27th	0.044	0.621	0.652	0.031	0.013	30%	27%	2%	8903	9590	18%	19%	1%
PBAT-T6 (2)		0.057	0.687	0.73	0.043	0.014	25%			10175		20%		
PBAT-T6 (3)		0.051	0.632	0.67	0.038	0.013	25%			9693		19%		

REPEAT OF BLANKS

	Time / hours	Mass before / g	boat / g	boat + film / g	mass after / g	mass change / g	% mass loss	Average mass loss	Std Dev	Mn	Average Mn	%	Average %	Std Dev
PBAT blank ref										42207	45477			
PBAT blank ref										44362				
PBAT blank ref										49863				
BLANK-PBAT-T1 (1)	4 days	0.049	0.674	0.723	0.049	6.93889E-17	0%	0%		44737	40940	98%		
BLANK-PBAT-T1 (2)		0.052	0.643	0.695	0.052	6.245E-17	0%		0%	37142		82%	90%	8%
BLANK-PBAT-T2 (1)	7 days	0.048	0.682	-		0.048	100%	100%		37142	38520	82%		
BLANK-PBAT-T2 (2)		0.049	0.61	-		0.049	100%			39121		86%		
BLANK-PBAT-T2 (3)		0.054	0.638	-		0.054	100%		0%	39296		86%	85%	2%
BLANK-PBAT-T3 (1)	14 days	0.047			0.0446	0.0024	5%	0%		26302	24508	58%		
BLANK-PBAT-T3 (2)		0.048			0.0504	-0.0024	-5%			23011		51%		
BLANK-PBAT-T3 (3)		0.053			0.0448	0.0082	15%		8%	24212		53%	54%	3%

5.4 Images of degraded films

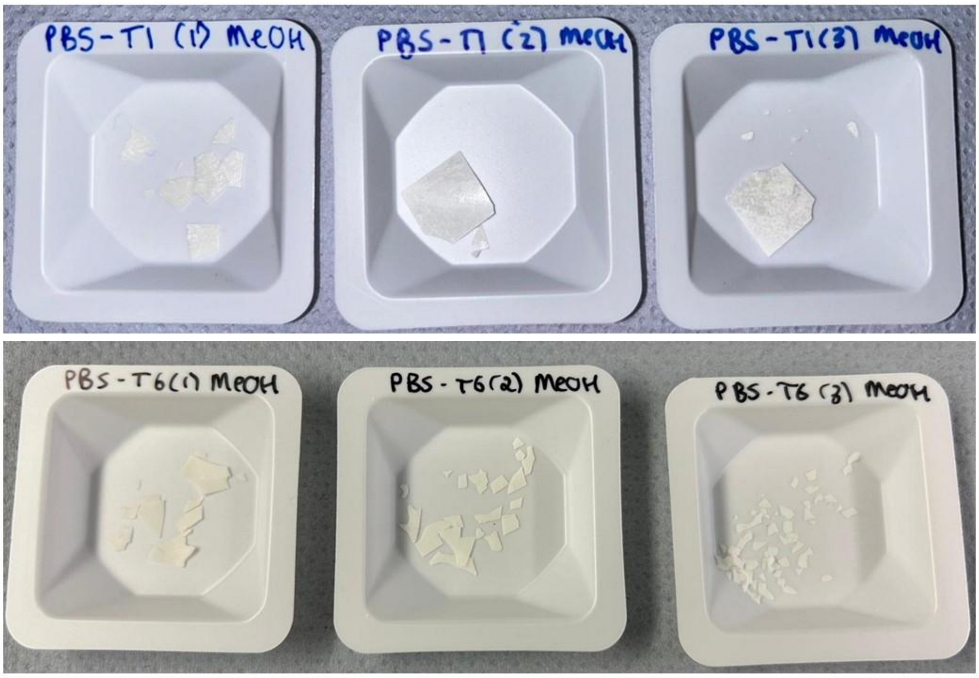

Figure S7: Example images of degraded PLA/$Zn(BAP)_2$ films.

6. $Zn(BAP)_2$ solubility in water

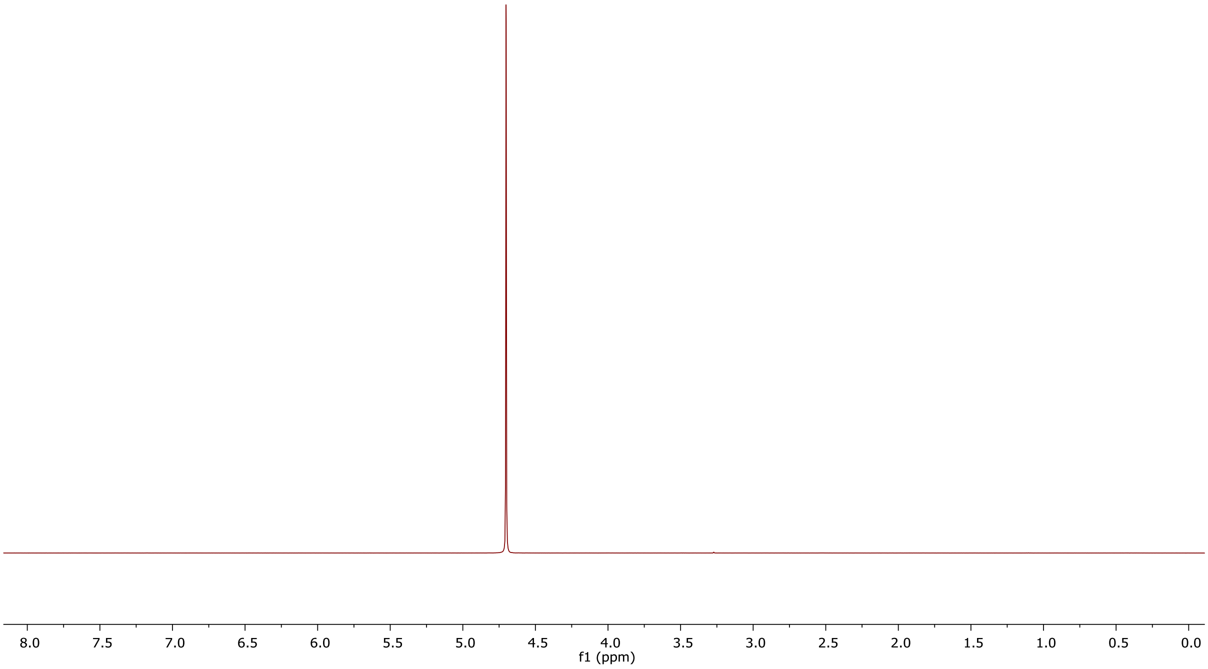

Figure S8: ^1H NMR spectrum (500 MHz, D_2O) of $Zn(BAP)_2$.
